# #Nicotineaddiction on TikTok: A quantitative content analysis of top-viewed posts

**DOI:** 10.18332/tid/151868

**Published:** 2022-08-05

**Authors:** Kristy L. Marynak, Meagan O. Robichaud, Tyler Puryear, Ryan D. Kennedy, Meghan B. Moran

**Affiliations:** 1 Department of Health, Behavior and Society, Bloomberg School of Public Health, Johns Hopkins University, Baltimore, United States

**Keywords:** social media, nicotine addiction, e-cigarettes

## Abstract

**INTRODUCTION:**

TikTok, the video-sharing app popular among youth, is a source of user-generated content about nicotine addiction with the potential to endorse or deter nicotine use among young viewers. We systematically analyzed content and themes of TikTok posts tagged #nicotineaddiction.

**METHODS:**

We conducted a quantitative content analysis of the visual and textual content of the 149 top-viewed English-language TikTok posts tagged #nicotineaddiction as of 1 March 2021. Posts were double-coded using a shared codebook, noting content creator characteristics, nicotine products featured, references to quitting, and overall themes of #nicotineaddiction expressed. We assessed the prevalence of post characteristics and themes overall and by apparent age of content creators (aged ≥21 years versus <21 years).

**RESULTS:**

The 149 posts analyzed received a mean and median of 62433 and 15800 likes, respectively. E-cigarettes were referenced or featured in 75% of posts; 58% featured a specific nicotine product brand, most commonly Puff Bar (23% of total) and JUUL (19%). Overall, 22% of posts mentioned quitting nicotine. The top themes of #nicotineaddiction expressed were physical or psychological consequences (e.g. withdrawal symptoms, 46%), physical or psychological benefits (e.g. tasting good, feeling ‘buzzed’, 28%), and social benefits (e.g. bonding with fellow users, 28%). Compared to those aged ≥21 years, posts by content creators likely <21 years (26%) less commonly mentioned quitting (p<0.01), had fewer followers (p<0.01), were more commonly from Canada (p<0.01) and less commonly from the US (p<0.01), and more commonly featured JUUL (p<0.05).

**CONCLUSIONS:**

While reaching a large and engaged audience, TikTok content creators suggest a range of benefits and consequences of nicotine addiction. Future research is warranted to examine this content’s potential to influence young people’s intentions to use or quit nicotine products.

## INTRODUCTION

Despite declines in cigarette smoking, use of any tobacco product, including e-cigarettes, is highly prevalent among US and Canadian youth and young adults. When asked about the use of any tobacco product in 2020, 23.2% of US high school students reported past 30-day use, and 18.2% of young adults aged 18– 24 years reported use ‘every day’ or ‘some days’^1,2^. In Canada, 18% of students in grades 7–12 reported past-30 day e-cigarette use in 2018–2019, and 6% of young adults aged 20–24 years were past-day e-cigarette users in 2017^3^. This is concerning because nicotine is addictive, harms brain development through age 25 years, and may prime adolescent brains for addiction to other drugs^4^.

The U.S. Food and Drug Administration (FDA) requires that e-cigarettes bear the statement: ‘WARNING: THIS PRODUCT CONTAINS NICOTINE. NICOTINE IS AN ADDICTIVE CHEMICAL’; and e-cigarettes sold in Canada warn ‘NICOTINE IS HIGHLY ADDICTIVE’. However, qualitative studies of adolescents reveal skepticism and uncertainty about nicotine addiction; some view addiction as a personal decision and a way to let one’s ‘body have fun’^5^. Whereas adults view messages about addiction more negatively, adolescents interpret them in both negative and positive terms, viewing addictive behaviors as ‘so good that people do not want to stop’^6^.

Young people are generally susceptible to peer influences and to social cues that normalize substance use, and media has been described as a ‘super peer’ because it exposes young people to information that they may not encounter through in-person social interactions^7-10^. Adolescents exposed to social media content that normalizes alcohol use are at greater risk for alcohol use^11^. Additionally, exposure to both advertising and user-generated content about e-cigarettes on social media during adolescence is associated with e-cigarette use^12,13^.

Social media platforms popular among young people such as TikTok, a short-form video-sharing app that is the fastest growing social media platform worldwide, reveal a proliferation of user-generated content about nicotine addiction^14–16^. TikTok posts tagged #nicotineaddiction had 117.4 million views on 1 March 2021. Although previous studies have explored content regarding vaping on TikTok^17^ and the Puff Bar e-cigarette brand specifically^16,18^, no studies have systematically explored content related to nicotine addiction on TikTok. As an estimated one-third of TikTok’s 49 million active users in the US are aged <14 years and TikTok is used by 44% of Canadian youth aged <18 years, these posts, which may be created by older cohorts of adolescents and young adults, are a potentially important source of information and social learning about nicotine for adolescents^14,19^. We systematically analyzed the content and themes of the most-viewed TikTok posts tagged #nicotineaddiction, including content related to tobacco cessation, given the content’s potential influence on social norms and youth use of tobacco products.

## METHODS

### Sample

The Johns Hopkins institutional review board determined that our study of publicly accessible social media content did not qualify as human subjects research. Data were collected in accordance with TikTok’s terms of service which prohibit downloading or copying of content^20^.

Posts tagged #nicotineaddiction were selected for analysis due to their focus on nicotine. As such, they are distinct from general posts about vaping or smoking, which may include use of cannabis or other substances. Additionally, given that hashtags are added by the content creators as part of their creative process, the #nicotineaddiction hashtag suggests that the creator may self-identify with nicotine addiction and/or have an interest in reaching audiences of people who identify with nicotine addiction.

We aimed to collect 100–150 posts to allow for saturation of thematic content and descriptive statistical analyses. We oversampled to account for anticipated removal of some posts from the platform, and exclusion of posts in languages other than English. Using the TikTok website unlinked to an account on a desktop browser, URLs and metadata (i.e. number of likes, comments, and followers) for the first 170 posts appearing in the feed for the hashtag #nicotineaddiction were collected during a single sitting on 1 March 2021. Given that no content was engaged with (i.e. liked, shared, or commented on) that might stimulate TikTok’s algorithm to push tailored content, the sample is expected to represent a common user’s experience initially exploring the #nicotineaddiction hashtag^21^. We verified that desktop content appeared in the same order as the mobile app by simultaneously comparing posts on a smartphone using a newly-downloaded TikTok app with an unused account. Posts appeared in the feed in descending order by number of likes. Posts in a language other than English (n=17) and those made private or removed prior to coding (n=4) were excluded from the analysis, yielding a final sample of 149 posts.

### Codebook

A codebook was developed to systematically collect demographic information for individuals posting and/or shown in posts, including country if listed, perceived age, perceived gender, and self-reported LGBTQ+ identification; nicotine product types, quantity, and brands shown or referenced in text; whether products were actively used or promoted; links to e-commerce sites to purchase products; references to quitting; use of humor; and theme(s) of addiction expressed (Table 1). Indicators of age <21 years included providing age in profile <21 years and/or context clues such as mentions of getting caught with nicotine products by parents or school administrators; setting, such as a school bathroom; appearance, such as signs of puberty or school uniforms; and admissions of attempted underage purchase of nicotine. Both deductive and inductive techniques were used to identify themes. Some potential themes were identified based on qualitative and theoretical literature around addiction^6,22,23^, and then approximately half of the sample was viewed to inductively identify additional addiction themes until apparent thematic saturation was reached (Table 2). When coding the full sample, coders could write in additional themes detected in posts.

**Table 1:** Characteristics of #nicotineaddiction TikTok posts, overall and by content creators’ perceived age, 1 March 2021

*Metadata*	*All posts (N=149)*	*Content creator likely <21 years (n=39) ^a^*	*Content creator likely ≥21 years (n=62)*	*p ^b^*
**Likes**				
Mean (SD)	62433 (193441)	63441 (103276)	83898 (286411)	0.67
Median	15800	21000	12800	-
**Comments**				
Mean (SD)	740 (2547)	821 (1429)	1011 (3761)	0.76
Median	119	126	93	-
Total number of content creators	106	33	27	-
**Followers per content creator**				
Mean (SD)	46028 (131090)	15482 (28754)	79247 (116848)	**0.004**
Median	6089	7625	21500	-
	***n (%)***	***n (%)***	***n (%)***	***p***
**Location of content creator, if listed**				
United States	50 (34)	9 (23)	34 (54)	**0.002**
Canada	10 (7)	7 (18)	0 (0)	**0.001**
Other location	3 (2)	2 (5)	0 (0)	0.15
**Perceived gender of people featured in post or profile picture**				
Male(s) only	70 (47)	16 (41)	32 (52)	0.32
Female(s) only	70 (47)	21 (54)	30 (48)	0.68
Gender unclear	7 (5)	1 (3)	0 (0)	0.39
**Likely age of people featured in post or profile picture** (years)				
<21^a^	39 (26)	39 (100)	0 (0)	-
≥21	62 (42)	0 (0)	62 (100)	-
Age unclear	47 (32)	-	-	-
**LGBTQ+ status indicated by content creator**	16 (11)	7 (18)	5 (8)	0.21
**Nicotine products actively used in post**	14 (9)	5 (13)	6 (10)	0.75
**Profile includes link to purchase nicotine products**	10 (7)	3 (8)	6 (10)	1
**Nicotine products shown or mentioned^c^**				
E-cigarettes	111 (75)	24 (62)	46 (74)	0.19
Puff Bar	34 (31)	7 (18)	12 (19)	1
JUUL	28 (25)	10 (26)	6 (10)	**0.049**
Other e-cigarette brands^d^	41 (37)	9 (23)	13 (21)	0.81
Cigarettes	14 (9)	5 (13)	5 (8)	0.50
Nicotine replacement therapy	7 (5)	3 (8)	4 (7)	1
Other nicotine product^e^	7 (5)	1 (2)	4 (7)	0.65
None shown or mentioned	24 (16)	12 (31)	7 (11)	**0.02**
**Estimated quantity of nicotine products shown**				
0	60 (40)	20 (51)	28 (45)	0.30
1	35 (24)	8 (21)	14 (23)	-
2–10	19 (13)	4 (10)	6 (10)	-
11–50	10 (7)	3 (8)	1 (2)	-
51–100	9 (6)	3 (8)	4 (7)	-
>100	16 (11)	1 (3)	9 (15)	-
**Tone**				
Humorous or ironic	133 (89)	36 (92)	55 (89)	0.74
**Quit references**				
Reference quit attempt (intention, in progress, or past)	32 (22)	4 (10)	21 (34)	**0.009**
No reference to quitting	117 (79)	35 (90)	41 (66)	-

a Indicators of age <21 years included providing age in profile under 21 (n=27, mean reported age 18.7) and/or context clues such as mentions of getting caught by parents or school administrators; setting, such as a school bathroom; appearance, such as signs of puberty or school uniforms; and admissions of attempted underage purchase of nicotine.

b P-value of t-test of difference in means for continuous variables (likes and comments) and Fisher's exact statistic for binary or categorical outcomes.

c Categories are not mutually exclusive. Percentage of e-cigarette brands were calculated from among e-cigarette-related posts (n=111).

d Other brands shown or mentioned in 2 or more posts include POP Bar (5), Stlth (5), Mr. Fog (4), Stig (4), Cuvie (3), Hyde (3), Suorin (3), VUSE (3), and Myst (2).

e Other nicotine products shown or mentioned include heated tobacco products (2), cigarillos (2), smokeless tobacco (1), hashish pipes (1), and oral nicotine pouches (1).

**Table 2:** Theme frequencies, coding descriptions, and examples from #nicotineaddiction TikTok posts as of 1 March 2021

*Theme of #nicotineaddiction ^a^*	*n (%)*	*Codebook description*	*Examples*
**Physical or psychological consequences**	68 (46)	Language or situations describing the negative effects of nicotine addiction or withdrawal on physical or emotional/psychological wellbeing, such as shaking or headaches; irritability, anxiety, depression, or preoccupation; impacts on lung or brain functioning, or injuries/adverse user experiences, including tasting bad	‘Never have I ever vape edition’ quiz asking person being filmed if they have ever ‘gotten winded’ climbing upstairs, gotten ‘nic sick’ or vomited from vaping, or lied when asked about e-cigarette use by a doctor, among other prompts Sketches or testimonial updates regarding nicotine withdrawal symptoms, some seriously depicting quit attempts and others in jest
**Physical or psychological benefits**	42 (28)	Mentions or depictions of positive impacts of nicotine use on physical or psychological state, including stress relief, calm, buzz, pleasure (including tasting good), escape, and/or concentration	Sketch of pretending to ‘hit everyone's nic’ and then suddenly feel a ‘buzz’ Prompt for viewers to ‘like when you see your favorite Puff Bar’, while showing a series of different flavors
**Social benefits**	41 (28)	Suggestions that nicotine addiction/use can be a basis for or strengthen social bonds between individuals (including through rituals), contribute to one's sense of belonging and identification with an imagined or actual community; and/or boost sexual attractiveness and social status/popularity	‘What your vape says about you’ memes showing different e-cigarette brands on a green screen and the content creators' opinions, such as that one who uses a certain brand is a ‘god’, or offering friendship for liking another brand Young person dancing in bathroom while text tells viewer to ‘hit’ their ‘nic’ right now and music plays ‘I just wanna party with you’
**Social consequences**	26 (18)	References to consequences that may arise between peers, family, or other community members, including alienation and conflicts, because of nicotine addiction	Comedic sketches of excuses one can use when someone asks to borrow their e-cigarette or conflicts arising when friends borrow e-cigarettes Girl singing about being on her guard when her boyfriend is ‘moody’ because he is out of nicotine
**Behavioral effects other than getting in trouble**	25 (17)	Language or situations depicting behavioral effects of nicotine addiction, such as lying to loved ones or progression to or co-use with other drugs, including different types of nicotine delivery (e.g. cigarettes), marijuana, and alcohol	List of things that have ‘happened to you at least once’ if you use e-cigarettes, including lying about having an e-cigarette device, being unable to go without nicotine, and trying to quit Addition of tags such as #alcoholic, #stoner; demonstration videos for how to refill or recharge (i.e. tamper with) disposable e-cigarettes
**Getting in trouble**	19 (13)	Mentions of or depictions of getting in (or risking getting in) trouble with parents, at school, with retailers, or with law enforcement for behaviors related to nicotine addiction	Vape shop employees or owners doing impressions of kids attempting to make underage purchases Young persons doing impressions of their nagging parents
**Quantity of involvement**	18 (12)	Demonstrations of or mentions of the quantity of involvement with nicotine use, indicated through references to an implied scale of size of addiction or through the amount of products previously consumed	‘Nicotine addiction check’ memes showing caches of often hundreds of used, disposable e-cigarette pods or devices arranged in color-coordinated rows, cascading into a bin. Often accompanied by a hip hop beat with percussive hacking and coughing sounds. Quizzes to see who has the ‘biggest nicotine addiction’
**Costs**	11 (7)	Language or situations that refer to money or time spent or wasted to support nicotine addiction	Sketch of deciding between spending remaining money on nicotine or alcohol while song plays ‘Maybe I need some rehab’ Sketches of frantic searches for missing e-cigarettes

a Coders could check all themes that apply.

### Procedures

Two trained coders independently coded a subsample of 25 posts to establish inter-rater reliability and inform codebook refinement. During March and April 2021, all 149 posts were independently double-coded using REDCap, a secure web-based data collection platform. Krippendorf’s alpha for all reported variables was 0.74^24^. A third coder resolved discrepancies.

### Analysis

Descriptive statistics were calculated overall and by perceived age of content creator. Differences in post characteristics, by content creator age, were assessed using t-tests of means for continuous variables (i.e. likes, comments, and followers) and Fisher’s exact tests of proportions for categorical variables. Statistical significance was assessed at a level of 2-sided p<0.05. Analyses were conducted using Stata version 16.1 in May 2021.

## RESULTS

As of 1 March 2021, the 149 top-viewed English-language TikTok posts tagged #nicotineaddiction had mean and median likes per post of 62433 and 15800, respectively (Table 1). The 106 content creators represented had a mean of 46059 and median of 6089 followers. Only 63 Tiktokers provided location information; overall, 34% listed the US, 7% listed Canada, and 2% listed another country (New Zealand, Bulgaria, and Switzerland). While age of content creators was undetermined for 32% of posts, 42% were created by individuals likely aged ≥21 years and 26% by individuals likely aged <21 years. Twenty-seven posts specified an age <21 years in the creator’s profile (mean: 18.7 years). Posts featured a similar proportion of perceived males and females, while 11% of posts included the content creator’s self-identification as LGBTQ+.

The most commonly featured nicotine-containing products were e-cigarettes (75%), followed by cigarettes (9%) and nicotine replacement therapy (NRT) (5%). Overall, 58% of posts showed or referenced a nicotine product brand other than NRT, most commonly Puff Bar (23%) and JUUL (19%). Additionally, 9% of posts promoted specific nicotine product devices and/or flavors, and 9% of posts showed a product being actively used. Five content creators of 10 posts (7% of total) included a URL link to an e-commerce site to purchase products, such as 812vapor.com or eCloudz.com; these content creators had a mean of 57220 followers.

Quitting nicotine products was referenced in 22% of posts, including in-progress quit attempts (13% of total), failed quit attempts (4%), and successful quits (3%). Compared to those with a perceived age of ≥21 years, content creators perceived to be <21 years less commonly mentioned quitting (p<0.01), had fewer followers (p<0.01), were more commonly from Canada (p<0.01) and less commonly from the US (p<0.01), and more commonly featured JUUL e-cigarettes (p<0.05).

Posts presented nicotine addiction in both positive and negative terms. The most prevalent themes of #nicotineaddiction, which are not mutually exclusive, were physical or psychological consequences, such as nicotine withdrawal (46%); physical or psychological benefits, such as generating a buzz or tasting good (28%); social benefits, such as bonding with other users (28%); and social consequences, such as peer irritability resulting from nicotine withdrawal (18%) (Table 2). Additional themes included behavioral effects other than getting in trouble, such as lying or co-using with other substances (18%); getting in trouble, including for underage possession (13%); demonstrations of one’s quantity of involvement with nicotine addiction, such as quizzes to compare who has the biggest nicotine addiction (12%); and costs, including time or money wasted or spent to support addiction (7%).

## DISCUSSION

Posts on TikTok reach a large and engaged audience with conflicting messages about physical, psychological, and social benefits and consequences of nicotine addiction. These data reflect organic discussions about nicotine addiction without researcher priming or instrument bias^25^. This social media content has the potential to shape attitudes about and intentions to use nicotine among TikTok’s very young user base.

The #nicotineaddiction hashtag may be viewed as a virtual tent under which users can find, connect with, and express their emerging identities^15^ as self-proclaimed nicotine ‘fiends’, recommend products and flavors, and share tips both for quitting or continuing to use nicotine. Content creators simultaneously express awareness of and disregard for the power of addiction and potential health consequences of nicotine and tobacco use, as in a satirical ode to nicotine addiction tagged ‘#imgonnadiesoon’, or a ‘never have I ever vape edition quiz’ prompting the person being filmed to indicate if they have ever been ‘nic sick’ or vomited from using e-cigarettes or have lied when asked by a doctor if they use cigarettes or e-cigarettes.

Addiction has historically conflicting popular meanings, involving both negative and positive connotations^22,23^. During the Early Modern Period in England (16th to 18th century), ‘addict’ meant ‘to attach’ to God, another person, or a behavior; such attachment could either be freely chosen or imposed^22^. In ‘Nicotine Addiction Check’ memes on TikTok, caches of used, disposable e-cigarettes or empty liquid refill pods are arranged in color-coordinated rows that cascade into a bin. These memes may signify one’s authentic involvement with vaping and nicotine use, aligning with the traditional meaning of addiction as attachment^22^.

Our finding that popular TikTok posts frequently espouse social, physical, and psychological benefits of nicotine addiction reinforce studies suggesting that prevention messaging and health warning labels for tobacco products focus on topics other than the addictive properties of nicotine^26-28^. For example, in focus group discussions with e-cigarette users and cigarette smokers, some rated the current FDA e-cigarette warning label as ineffective because ‘everyone already knows nicotine is addictive’; and nicotine in e-cigarettes is ‘part of the appeal’^28^. Pediatric healthcare providers should be aware that patients may be exposed to social media content that starkly contrasts current prevention messages. Providers and public health practitioners may emphasize alternative messages that have been found to be effective in deterring young people’s intentions to use e-cigarettes, including tobacco industry manipulation of young people; exposures to harmful chemicals in e-cigarettes; and respiratory harms of e-cigarette use^29-32^. Given the prevalence of posts depicting negative physical or psychological effects of nicotine addiction, efforts are warranted to investigate the effectiveness of messages that elaborate on specific negative consequences of nicotine addiction, such as stress caused by withdrawal.

TikTok posts frequently blur the line between commercial and non-commercial content, with some linking to e-commerce sites and/or endorsing brands without disclosing sponsorships. For example, in ‘What your vape says about you’ memes, self-professed experts align e-cigarette brands with user typologies, suggesting that specific brands may enhance or diminish social status. The tobacco industry has exploited the unique vulnerability of young people to tobacco addiction and social influences through product design and marketing^8,33^. Previously internal documents reveal the tobacco industry’s targeting of ‘pre-smokers’ and ‘learners’, who expect cigarette smoking to result in ‘group identification – participating, sharing, conforming, etc.’ and ‘self-image enhancement – identification with valued persons, daring, sophisticated, free to choose’^33,34^. Efforts are warranted to increase the transparency of sponsored TikTok content and establish age restrictions on access to branded and/or tobacco-related content.

### Strengths and limitations

Our study had several strengths, including the selection of a social media platform that is popular among youth and young adults, who are also a population of concern with regard to e-cigarette use. Additionally, since the analyzed content is user-generated, there is no researcher priming or instrument bias in the data collection process, as there might be with survey data; further, the content creators’ deliberate addition of the #nicotineaddiction hashtag provides a sound basis for exploring themes related to addiction. Thus, it is likely that those who use nicotine more freely convey any perceived benefits of nicotine addiction. Additionally, while most of the content analyzed was related to e-cigarettes, the selection of the #nicotineaddiction hashtag included content on other tobacco and nicotine products, allowing for an exploration of nicotine addiction as conceptualized across the landscape of tobacco products. Finally, our novel method of collecting URLs for analysis adheres to TikTok’s restrictive terms of service and could be replicated for other studies.

We note at least three limitations. First, demographic information and location of content creators could not be verified. Second, given that our study was limited to posts tagged #nicotineaddiction, additional posts related to the topic but without the hashtag were not explored; and the convenience sample of top-viewed posts is not necessarily generalizable to all content from less-viewed posts with the same or similar tags. Third, our analysis reflects the environment at one point in time amid a dynamic social media landscape. Nevertheless, social media content analyses can complement surveys and in-depth qualitative research to inform clinical, educational, and regulatory interventions. Specifically, interviews could provide additional insight into the content creators’ perceptions and intent when developing and tagging posts.

## CONCLUSIONS

TikTok posts tagged #nicotineaddiction suggest a range of benefits and consequences of nicotine addiction. This content may undermine the effectiveness of prevention messages solely focused on problematizing the addictive properties of nicotine. Future research should examine the effect of exposure to content on viewers’ attitudes about nicotine products and intentions to initiate, continue, or quit nicotine use.

## Data Availability

The data supporting this research are available from the authors on reasonable request.
